# Clinical Report from Medical Wards of Mercy Hospital

**Published:** 1868-11

**Authors:** N. S. Davis

**Affiliations:** Professor of Clinical Medicine, etc.


### The Clinique.

### CLINICAL REPORT FROM MEDICAL WARDS OF MERCY HOSPITAL.

Service of N. S. DAVIS, M.D., Professor of Clinical Medicine, etc. Reported by W. A. BARSTOW.

### PROBABLE SOFTENING OF THE BRAIN.

GENTLEMEN:—The case before you is one of interest, more especially in regard to its pathology and diagnosis. The patient is a native of Ireland, about 45 years of age, naturally muscular and strong; by occupation a laborer.

As you look at him in bed, you readily recognize a vacant, staring expression of countenance; a dorsal position, with limbs extended; not a tremulous, but an unsteady and backward movement of the hands; and in answer to my questions, you notice his speech is slow, hesitating, and frequently stops in the middle of a sentence, apparently losing the connection of thought. His mind often wanders, and his sleep is disturbed. He complains of no pain, and exhibits no sign of fever, not even increased heat in his head. The pupils are slightly dilated, and the vessels of the conjunctiva free from congestion.

He protrudes his tongue readily, giving it a narrow and pointed shape, and you see on its surface a thick, moist coat, particularly along its middle line. You find his skin soft, temperature natural, and sensibility good.

While lying in bed he can move all his limbs, and in any desired direction; and yet he can neither get up, nor stand erect, nor walk. He has only a partial control over the sphincters; both urine and fæces being passed in bed. His pulse is soft and rather slow, but not intermitting; and when awake his respirations are nearly natural.

This man was admitted into the hospital only two days since, and his history, so far as I can obtain it, is as follows:—About six months since his friends began to notice slight indications of failure in his mental faculties, and some unsteadiness in his walk. These indications gradually increased, without the supervention of any sudden or severe sickness, until he has arrived at the entirely helpless condition, mentally and physically, that you see before you. For several years previous to the supervention of his present sickness, he had been addicted to the intemperate use of alcoholic drinks; and the same were continued for two or three months after his health began to fail.

With this review of the history and present symptoms, where is the seat of disease, and what the nature of the pathological changes which have taken place? The inability to maintain an upright position or to control the sphincters of the bladder and rectum, taken in connection with the impaired state of the mental faculties, show plainly that the seat of disease is within the cranium.

We may have inability to walk, with loss of control over the sphincters from disease of the spinal cord or its membranes; but in such cases the mental operations would not be impaired. But conceding the seat of disease to be the brain or its coverings, what is the nature of such disease? Is it a chronic inflammation of the cerebral substance? Or, is it a slow atrophy from defective nutrition? Or, again, can it be a mere functional derangement, consisting of impaired cerebral sensibility? To answer these questions reliably requires close examination of the patient, and an accurate knowledge of the symptoms that distinguish one pathological condition of the brain from another. Inflammation, either acute or chronic, involving the membranes or convolutions of the brain, causes increased heat; pain; restlessness; acuteness of sensibility; with positive mental derangement in the first stage; followed by effusion; paralysis; and coma, or continued insanity, in the second. If the inflammation involve the interior of the brain, there may be less evidence of mental derangement, but there will still be pain; indisposition to move the head; increased temperature; altered pupils; and more or less rigidity of the voluntary muscles, either of the neck or extremities.

At no period in the history of the case before us were any of these symptoms characteristic of cerebral inflammation present.

On the contrary, the head has been free from heat or pain, and the muscular system more and more flaccid and feeble in contractility, from the commencement of ill-health to the present time.

Mere functional disturbances of the brain are usually variable in the symptoms, and rarely cause a steadily progressive loss of flesh or of muscular strength. But when the nutrition of the brain has been impaired, by the long continuance of slowly-acting causes, and the texture begins to soften or atrophy from deficiency of atoms, the symptoms are those of simple impairment of function.

The patient is not, at first, either delirious or paralyzed. He becomes forgetful; is unable to maintain continuous thought and expression, often losing the thought in the middle of a sentence; while his gait becomes unsteady, and all his muscular movements enfeebled. The symptoms thus begun generally increase steadily, until the mental manifestations become simple, imperfect, and sometimes incoherent; and the muscular action so impaired as to render the patient incapable of walking, or even of maintaining the erect position. Only a few weeks since, a young man occupied a bed in this ward, who had been employed as a clerk in a grocery store, and had been regarded as a reliable and correct young man. He was first noticed to be despondent, without any known cause. Soon he became forgetful; then slow in his movements, and after some weeks wholly incapable of doing business. When admitted into the hospital, his face was pale; expression downcast and vacant; skin cool; pulse slow and soft; bowels inactive; and entire loss of all disposition to either mental or physical exertion. It was difficult to induce him to answer a question; and when he did his voice was weak, and his expression slow and interrupted. He neither asked for food or drink, but would sit in a chair or lie in bed from morning until night almost motionless. It was necessary to feed him in the same manner as a young child; and, at suitable intervals, call his attention to the necessity of evacuating the bladder and rectum. He complained of little or no pain, exhibited no spasmodic action of muscles or rigidity, but sometimes, especially in the night, under some mental hallucination, he would attempt to wander about the house.

After observing him for some time, and trying without benefit several items of treatment, I became satisfied that the condition of the brain was that of anæmia and deficient nutrition, but not yet advanced to the degree of actual softening or disintegration. He was consequently put upon the use of syrup of pyrophosphate of iron, in doses of a teaspoonful three or four times a day, a diet of plain, nutritious food, such as milk, bread, rice, tender meats, etc.; and the use of an electro-magnetic battery once or twice every day. He was also encouraged to make efforts at walking, and, as soon as possible, to get out in the open air every day.

This course of treatment was carried out faithfully several weeks, and resulted in a slow recovery of the patient, who is now at his previous occupation. All the symptoms of the case before you have been, and now are, of such a character as to indicate a similar arrest of nutrition of the brain, more especially of the internal parts. But in this case the morbid process has probably continued until the texture has become so far changed as to be unable to perform its functions. In other words, some parts of the brain are in a state of *ramollissement*, or softening. If so, the prognosis is exceedingly unfavorable; and there would be little benefit from any kind of medical treatment. So long, however, as we cannot know positively that cerebral disorganization exists, it is our duty to prescribe such treatment as would be most likely to restore the patient, if the structural changes had not reached a stage incapable of repair. The indications are to increase the sensibility and activity of the mucous centres, thereby restoring more steady and efficient muscular action, especially in the involuntary muscles, and to improve the nutrition of the brain. Probably small doses of strychnine would fill the first indication, and some one of the phosphatic salts of iron the second, as well as any remedies that we could select. Hence, we will direct the following prescription:—

℞. Strychnine,-------------------------- 1 gr.

Nitric Acid, -------------------------- ʒj.

Simple Syrup, ------------------------- ℥j.

Water,--------------------------------- ℥ij.

Mix, and give a teaspoonful every six hours, diluted with sweetened water.

Also, one teaspoonful of the pyrophosphate of iron every six hours; making the medicines come alternately three hours apart. We will have him fed regularly with milk, bread, rice, etc.

The result you will be able to learn at a subsequent clinic.

